# Editorial for Special Issue “Materials Frontiers for Solid Oxide Fuel Cells (SOFCs): Structure–Performance Correlation”

**DOI:** 10.3390/ma18235267

**Published:** 2025-11-21

**Authors:** Alessandro Dell’Era, Enrico Bocci

**Affiliations:** 1 Department of Basic and Applied Sciences for Engineering, Sapienza University, Via del Castro Laurenziano 7, 00161 Rome, Italy; 2 Department of Engineering Science, Marconi University, Via Plinio 44, 00193 Rome, Italy

In recent years, novel technologies have been proposed to produce energy, such as fuel cell systems. In general, fuel cells have some advantages, such as high efficiency (almost 75–85%) [1,2], appropriateness for stationary and mobile applications [3,4], as well as very low or no CO_2_ emissions [5]. Fuel cells work at both low and high temperatures on the base of their electrolyte, and solid oxide fuel cells (SOFCs) in particular demonstrate a high potentiality [6], with a total efficiency of up to 85% if heat cogeneration is taken into account. One of the main issues in fuel cell technology is catalyst degradation, which operates at high temperatures, converting several types of fuels into electricity with high efficiency and minimizing the deterioration of electro-catalysts. Six manuscripts have been published in this Special Issue covering topics regarding the synthesis, characterization, and study of advanced materials for the optimization of SOFC/SOEC energy systems, which can be used to produce electricity or hydrogen with high efficiency.

In one paper (contribution 1), Yan et al. investigated the optimization of the tape casting synthesis process of porous metallic support for SOFC electrodes. In particular, they analyzed how the diameter size of the pore-formers and their quantity in terms of volume fraction influence the properties of 430 L stainless steel substrates made by tape casting and sintering. The authors aimed to optimize the metal support to have good gas permeability, adequate porosity, and a surface that allows the deposition of the subsequent layers via plasma spray, without excessively large pores causing defects. This support material is made of fine 430 L steel powder with the addition of PMMA (polymethyl methacrylate) beads as pore-formers. The pore former sizes used were 20 µm, 40 µm, 60 µm, 90 µm, designated PF20, PF40, PF60, and PF90, and different volume fractions of the pore former were used. All the supports were characterized in terms of final porosity, open pore size, gas permeability, surface roughness, thickness of the oxide layer after oxidation, and mass gain after oxidation in air at 900 °C for 24 h. The pore former with a diameter of 60 µm (PF60) and volume fraction of approximately 45 vol% provided the best results, obtaining a gas permeability of approximately 3.11 × 10^−13^ m^2^, an average open pore size of approximately 45.90 µm. The largest pore detected on the surface had a diameter of 49 µm, considered acceptable for the subsequent layer deposition via plasma spray. In fact, too few and too large pores would cause defects. In practice, it has been found that using small pore-formers (PF20 = 20 µm) results in lower porosity, greater shrinkage during sintering, and lower permeability. With larger pore-formers (PF90 = 90 µm), more isolated pores are obtained, but this also causes lower connectivity of the pore network and slightly lower permeability compared to PF60. The porosity perimeter/steel ratio decreases with increasing pore-former size, ensuring a smaller internal exposed metal area and consequently a lower unwanted oxidation reaction. However, on the surface after 900 °C for 24 h in air, the oxide thickness is approximately 2 µm on all samples, indicating that the metal substrate does not show any problems with warping, cracking, excessive oxidation, or structural collapse. Compared to an existing commercial substrate with a similar porosity perimeter to steel area ratio, the new substrate (PF60-45 vol%) showed approximately 1.6 times higher porosity and approximately 3 times higher gas permeability. In conclusion, the fact that PF60/45 vol% was the most effective substrate implies that there is a balance to be found between a pore size large enough to ensure good permeability, but not so large as to compromise electrolyte deposition and sealing, and a volume fraction sufficient to create a pore network, but not so high as to compromise mechanical integrity or the surface. Finally, the support with controlled porosity offers the possibility of using the plasma deposition technique for subsequent layer deposition, with advantages in terms of time and costs compared to traditional ceramic methods.

In another study (contribution 2), C.M. Ruse et al. emphasize that, in SOFC devices, the anode microstructure, in terms of ceramic-metallic composition, material distribution, and porosity, plays a crucial role, determining and influencing the number of active reaction sites, as well as electronic and ionic conductivity, and gas transport to the active sites. The aim was to quantify the anode microstructure characteristics of SOFCs and relate them to their electrochemical performance. Through a sintering process, electrolyte–anode bilayers were prepared consisting of a Ni/YSZ anode on a support and an YSZ (Y_0.16_Zr_0.84_O_1.92_) electrolyte. To improve electrochemical compatibility, an interlayer consisting of Gd_0.2_Ce_0.8_O_1.9_ was screen-printed on the electrolyte and sintered at 1200 °C for 2 h. Instead, La_0.6_Sr_0.4_Co_0.2_Fe_0.8_O_3_, with an area of approximately 2 cm^2^, was sintered at 1080 °C for 30 min and used as cathode. In particular, two different temperatures were used for the sintering process of the electrolyte–anode bilayers to evaluate how this variable affects the distribution and quantity of the ceramic and metallic phases, consisting of YSZ and Ni, respectively. The total porosity and the connected porosity, related to the permeability to hydrogen flow, were also evaluated, along with the length of the triple-phase boundary (TPB), i.e., the interface where the gas, the ceramic material (ionic conductor), and the metallic material (electronic conductor) meet. All these characteristics, in fact, influence electrochemical performance in terms of power density, cell resistance, etc. A 3D focused ion beam scanning electron microscopy (FIB-SEM) technique was used, followed by a 3D digital reconstruction. The cell sintered at 1365 °C (T2) showed a more favorable microstructure, namely, a more balanced ratio between the ceramic phase (41 vol% YSZ) and the metallic phase (45 vol% Ni), with a total porosity of approximately 14.9 vol% and a connected porosity of 10.65 vol%. The cell sintered at 1450 °C (T1) showed a less favorable microstructure (55.4 vol% YSZ and 22.9 vol% Ni), with a total porosity of approximately 21.7 vol% and a connected porosity of 16.38 vol%. Despite differences in connected porosity, the permeability to hydrogen flow is similar (approximately 8.8 × 10^−3^ md for T1 and 8.0 × 10^−3^ md for T2). Therefore, this result shows that gas transport is not limiting and it is not responsible for the different electrochemical performances. Instead, the TPB length per unit volume (TPB density) was approximately 3.92 µm^−2^ for T2 and 1.96 µm^−2^ for T1. Therefore, the cell obtained with a lower sintering temperature (T2) essentially had double the density of the TPB active sites. From an electrochemical point of view, the T2 cell showed a higher power density and a lower total specific resistance, equal to approximately 0.86 W/cm^2^ and 0.69 Ω cm^2^, respectively, while the cell obtained at T1 presented a power density equal to 0.19 W/cm^2^ and a specific resistance of approximately 4.88 Ω cm^2^. In their conclusions, the authors highlight how the sintering process, and, in particular, the temperature, is a fundamental parameter for anode microstructure. Excessively high temperatures can lead to unfavorable material distribution, a reduction in the TPB length, and a deterioration in electrochemical performance. Furthermore, the work demonstrates that by using 3D characterization techniques such as FIB-SEM together with 3D digital reconstruction, it is possible to obtain a precise quantitative description of the actual microstructure (rather than hypothetical models) and obtain a direct correlation with electrochemical performances.

For the proper operation of SOFCs/SOECs, the channels of the electrodes through which the gases diffuse must have an appropriate aspect ratio, because it influences various operational phenomena such as pressure drops, flow distribution and uniformity in the distribution of the active gas along the electrode, mass exchange (transport of reactants towards the active surface), gas diffusion within the channel, and finally the contact between the gas and electrode surface, which, in turn, influences the electrochemical reaction. In their work, Yao et al. (contribution 3) aimed to understand how the geometry of the flow channel, specifically the aspect ratio (channel width/height ratio), affects the performance of a solid oxide electrolysis cell (SOEC). They developed three-dimensional models for channels with constant cross-sectional area but varying aspect ratios to study this effect. They developed a 3D model that encompasses various physical phenomena (multi-physics) such as mass transport, heat transport, electrochemistry, and fluid dynamics within the channels designed to distribute gas and allow it to reach the active sites. Specifically, they studied how varying the aspect ratio produces different results under the same conditions. The aspect ratio values used were 0.25 (narrow and tall), 1 (square), and 4 (wide and flat), keeping the cross-sectional area constant. The simulations were validated with experimental data obtained in the literature to verify the model’s reliability. It has been determined that channels with a very low (0.25) or very high (4) aspect ratio show a greater pressure drop, while the one with an aspect ratio equal to 1 has the lowest pressure drop. Regarding gas distribution, it has been shown that the mole fraction of hydrogen near the cathode surface tends to decrease as the aspect ratio increases. In fact, for a high aspect ratio (e.g., “wide and flat” channel, ratio = 4) the geometry favors better diffusion, so the hydrogen produced is diluted more rapidly with the incoming vapor. These factors also influence electrochemical performance, since with higher aspect ratios, better performance is obtained in terms of current density due to better mass transport caused by a shorter diffusion path. With an aspect ratio equal to 0.25, acceptable performances are obtained at low current densities, but they worsen when the required current density increases. In conclusion, however, an aspect ratio value equal to 1 was considered the most balanced solution, through which a good compromise could be obtained between pressure drop, mass transport, manufacturing complexity, and variable operating conditions.

In addition to the structure and design of an electrochemical cell, the materials also play a crucial role in the desired performances [7,8]. In their study, Cascos et al. (contribution 4) researched a series of SrMo_1−x_Mg_x_O_3−δ_ oxides (perovskites) with x = 0.1; 0.2 as anode materials for solid oxide fuel cells (SOFCs). These materials are characterized by mixed ion-electron conductions (MIECs) due to the introduction of the Mg^2+^ ion in place of Mo^4+^, which generates oxygen vacancies and improves ionic conductivity. It is known that during sintering or exposure to an oxidizing environment, the perovskite SrMo_1−x_Mg_x_O_3−δ_ phase oxidizes, transforming into a scheelite-type SrMo_1−x_Mg_x_O_4−δ_ phase. In particular, the authors examined the structural mechanism of the topotactic (oxidation/reduction) transformation between these two phases through X-ray diffraction (XRD) and neutron scattering (NPD) characterizations. They observed that the perovskite → scheelite transformation can occur at moderate temperatures of around 400 °C in air and without the formation of evident intermediate phases, implying a good topotactic relationship between the structures. The perovskite phase is cubic (space group Pm 3 m) while the scheelite is tetragonal (space group I4_1_/a), and the transformation involves the insertion of additional oxygen and changes in the coordination lattices of the Mo/Mg ions. In the perovskite, regular MoO_6_ octahedra share vertices, producing a compact and denser structure. Oxygen vacancies are still present but uniformly distributed, favoring ionic and electronic conduction. Scheelite, on the other hand, features a tetrahedral MoO_4_ coordination, but the tetrahedra are separated from each other, producing a more open structure that favors the mobility of oxygen ions and the reversibility of the redox cycle. In practice, the scheelite phase can incorporate extra oxygen, while the perovskite can release oxygen without collapsing the structure. Furthermore, the authors verified that both phases (oxidized and reduced) present significant oxygen vacancies, thus ensuring good ion conduction and therefore good behavior as an MIEC material. In their conclusions, the authors suggest that this reversibility and structural stability during oxidation/reduction are important for using these materials as anode in SOFCs, because they reduce the risk of cracking, delamination, or structural degradation during redox cycles.

Moreover, SrFeO_3−x_ has been studied as a material for use in SOFC applications, but it has some disadvantages. SrFeO_3−x_ tends to be thermodynamically unstable in highly reducing environments and can transform into non-conductive phases, such as SrFeO_2_, reducing performance. The composition therefore changes with temperature and environmental conditions (oxidizing vs. reducing), causing unwanted structural variations. The conductive and electrocatalytic properties of SrFeO_3−x_ depend strongly on the value of x, which represents the number of oxygen vacancies. Maintaining stable stoichiometry during cell operation, however, is difficult. At different values of x, the material can undergo a transition from a cubic perovskite phase to an orthorhombic phase. This phenomenon, combined with thermal expansion, can cause mechanical stress, delamination, and cracking during thermal cycling. Furthermore, SrFeO_3−x_ can react with electrolytes such as YSZ (yttria-stabilized ZrO_2_), forming insulating secondary phases, such as SrZrO_3_ or Fe_2_O_3_, degrading the electrode–electrolyte contact and increasing interfacial resistance.

In this context, A.I. Ivanov et al. (contribution 5) synthesized and characterized the iron-based perovskite SrFeO_3−δ_ as an electrode material in SOFC/SOEC systems. For the synthesis, they used the citrate–nitrate method, with final sintering in air. In particular, the authors wanted to study how, as a function of temperature and oxygen partial pressure (p(O_2_)), the substitution of part of Fe with V influences the presence of oxygen vacancies (δ), the electrical conductivity of the material, chemical expansion and finally, the electrode properties. The specific composition examined is SrFe_0_._9_V_0_._1_O_3−δ_, i.e., with 10% of Fe replaced by V. A structural analysis (XRD) was carried out that revealed a cubic perovskite phase (space group Pm$\bar{3}$m), while the concentration of oxygen vacancies was measured by coulometric titration in a wide range of p(O_2_) (from 10^−21^ to 0.5 atm) and T (from 1023 to 1223 K). It has emerged that the introduction of V reduces the oxygen deficiency (*δ*) compared to the undoped material, and lowers the average oxidation of the iron ion (a smaller amount of Fe^4+^). Consequently, the electrical conductivity at 923 K of SrFe_0_._9_V_0_._1_O_3−δ_ is approximately 14% lower than that of SrFeO_3−δ_ because there are fewer charge carriers (fewer p-type electron holes) and because V^5+^ immobilizes part of the structure, causing the oxygen vacancies to move with greater difficulty. Despite this, a lower *δ* produces greater chemical stability in the material, causing less degradation, especially in oxidative conditions. Furthermore, with vanadium, since the structure is more immobilized, it is less subject to chemical expansion and therefore the material resists thermal and mechanical stress better during on–off cycles of the cell. From the electrochemical point of view the porous electrode shows better activity in SOEC mode than in SOFC mode, the specific polarization resistance under air conditions at 1173 K is 0.34 Ω cm^2^ in open circuit conditions, and the exchange current density *i*_0_ is calculated as equal to 35 ± 5 mA/cm^2^. In conclusion, replacing Fe with V improves some critical properties, namely, it reduces oxygen deficiency, reduces chemical expansion that causes mechanical degradation, and improves thermal compatibility with the electrolyte compared to undoped SrFeO_3−δ_. On the other hand, however, it causes a reduction in conductivity and stability under highly reducing conditions that are not yet perfect for use in a complete SOFC anode. However, in general the study provides a good basis for the design of electrodes for solid oxide cells, showing how the non-stoichiometric chemistry of oxygen influences transport, expansion, and electrochemical activity.

Finally, in the final article, S.B. You et al. (contribution 6) studied a material often used in SOFC technology for another electrochemical system—the lithium battery. This study investigates the diffusion characteristics of lithium ions in lithium-containing and lithium-free bismuth oxide crystals, aiming to explore their potential as solid electrolytes for next-generation lithium-ion batteries. Although bismuth oxide has been widely used as a solid electrolyte in fuel cells, its suitability for lithium-ion battery applications remains unexplored. Using molecular dynamics simulations, the authors analyzed the diffusion behavior of lithium ions in two distinct lithium-containing bismuth oxide crystals with layered and non-layered structures, as well as in four lithium-free bismuth oxide phases. The layered structure was shown to exhibit a simpler and more organized diffusion path than the complex, bottleneck-like paths of the non-layered structure, resulting in higher lithium-ion diffusivity. For lithium-free bismuth oxide phases, diffusion coefficients vary significantly depending on structural characteristics, with the highest diffusion coefficient observed in the phase with the lowest void fraction. A remarkable inverse relationship between void fraction and lithium-ion diffusion efficiency highlights the importance of structural design in enhancing ion transport. This study provides valuable insights into lithium-ion diffusion mechanisms in bismuth oxide systems and offers strategic guidance for the design of high-performance solid electrolytes, contributing to the advancement of solid-state battery and fuel cell technologies.
